# A machine learning approach for ranking clusters of docked protein‐protein complexes by pairwise cluster comparison

**DOI:** 10.1002/prot.25218

**Published:** 2017-01-20

**Authors:** Erik Pfeiffenberger, Raphael A.G. Chaleil, Iain H. Moal, Paul A. Bates

**Affiliations:** ^1^ Biomolecular Modelling LaboratoryThe Francis Crick InstituteLondonNW1 1ATUK; ^2^ European Molecular Biology LaboratoryEuropean Bioinformatics Institute, Wellcome Trust Genome Campus, HinxtonCambridgeCB10 1SDUK

**Keywords:** CAPRI, protein complex prediction, scoring, machine learning

## Abstract

Reliable identification of near‐native poses of docked protein–protein complexes is still an unsolved problem. The intrinsic heterogeneity of protein–protein interactions is challenging for traditional biophysical or knowledge based potentials and the identification of many false positive binding sites is not unusual. Often, ranking protocols are based on initial clustering of docked poses followed by the application of an energy function to rank each cluster according to its lowest energy member. Here, we present an approach of cluster ranking based not only on one molecular descriptor (e.g., an energy function) but also employing a large number of descriptors that are integrated in a machine learning model, whereby, an extremely randomized tree classifier based on 109 molecular descriptors is trained. The protocol is based on first locally enriching clusters with additional poses, the clusters are then characterized using features describing the distribution of molecular descriptors within the cluster, which are combined into a pairwise cluster comparison model to discriminate near‐native from incorrect clusters. The results show that our approach is able to identify clusters containing near‐native protein–protein complexes. In addition, we present an analysis of the descriptors with respect to their power to discriminate near native from incorrect clusters and how data transformations and recursive feature elimination can improve the ranking performance. Proteins 2017; 85:528–543. © 2016 Wiley Periodicals, Inc.

## INTRODCUTION

Specific protein–protein interactions are key to most cellular functions, ranging from effective signal transduction of environmental conditions to the nucleus to modulation of cell‐cell interactions and efficient regulation of metabolic processes.^1^, ^2^, ^3^

Experimental determination of such interactions at the atomic level has improved our knowledge of these cellular processes but there are still many protein–protein interactions for which atomic level information is not available. Given the relatively slow accumulation of experimental data, computational protein docking is seen as the method of choice to complete the protein interaction space.^4^ However, there are two intertwined problems to solve before these methods can be routinely employed. The first is to develop methods to efficiently sample the conformational space of the interacting proteins,^5^, ^6^ perhaps aided by experimental data.^7^ The second is to be able to effectively rank docked poses, from typically thousands generated by current docking algorithms,^8^ to identify docking ensembles (clusters), or single docked poses that resemble native‐like binding. Here we focus on the latter problem and develop a machine learning protocol to rank clustered docked poses in order locate the cluster, if present, that most likely represents the bound state. Compared to approaches in which single structures are ranked, this cluster‐centric approach better reflects the interaction as an ensemble of conformational arrangements which, in some cases, are so diffuse that no single crystal structure snapshot gives a reliable representation of the bound state.^9^

The importance of a cluster based ranking method with high accuracy for its top ranked solutions is especially significant when association and disassociation rates are computed for the binding mode represented in each cluster. Highly accurate methods use extensive molecular dynamics (MD) simulations and often require ns to converge^10^, ^11^ and therefore a reduced solution space to test is the only tractable approach. In addition, computationally expensive refinement or relaxation methods based on conformational sampling from MD simulations^12^ also benefits from a reduced solution space. These are often required to correctly model conformational transitions from unbound to bound.^13^

Even though there have been a number of individual potentials developed for the identification of protein–protein interactions all of them suffer from false positive identifications of binding modes whereby incorrect solutions are ranked highly.

Here we present a novel method that combines a statistical learning from pairwise cluster comparisons based on a large number of molecular descriptors important for protein–protein interaction, with localized cluster enrichment with SwarmDock,^14^, ^15^ to discriminate near‐native from incorrect clusters. To the authors knowledge, this method of ranking clustered docked poses is the first of its kind. However, machine learning methods, such as PROCOS,^16^ which is based on training a support vector machine, has been applied to classify docked complexes as native‐like or false. Compared to our method, PROCOS makes use of a very limited number of molecular descriptors (i.e., electrostatic energy, van der Waals force and a knowledge based pair‐potential), does not exploit the information of the cluster environment and a pairwise cluster comparison to train a model that is crucial to our method. Further applications of machine learning in the field of protein–protein interactions have been applied to predict binding affinities such as ΔG^17^, ^18^, ^19^ and disassociation rates such as k_off_.^20^, ^21^

The benchmark of our method is based on the score_set decoy set of docked protein–protein complexes.^22^ This set of decoys originates from Critical Assessment of Prediction of Interactions (CAPRI) experiment where protein–protein docking and ranking methods are evaluated in blind predictions.^23^, ^24^

In the following sections, we first outline the challenge for a machine learning protocol for ranking clusters of docked poses and explain the unique properties and advantage of our solution. Furthermore, we present an analysis of the 109 molecular descriptors with respect to their power to discriminate near native from incorrect clusters and their co‐linearity. We then show that a reduced set of features based on these 109 molecular descriptors is beneficial for ranking performance and that dimensionality reduction and feature space transformations with methods such as principal component analysis (PCA) or factor analysis (FA) can improve the top 1 and top 5 ranking. Overall, our best approach based on a reduced set of features was able to rank the near‐native cluster that contains the model with the lowest ligand root mean square deviation (LRMSD), with an average rank of 3.5.

## METHODS

### Overview

**Figure 1:**
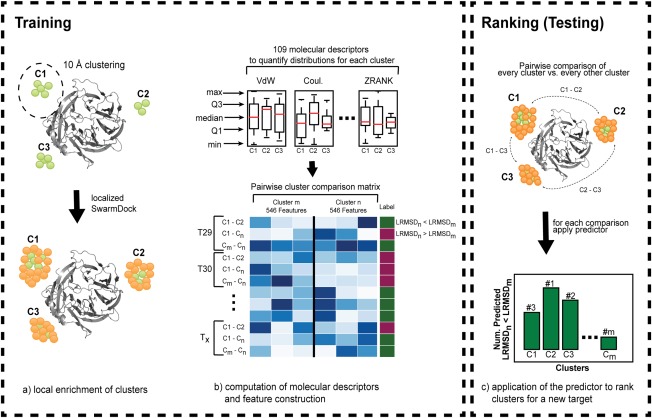
Schematic overview of the method. In a first step (a) decoys are clustered with a 10 Å cutoff and clusters are enriched with additional solutions with localized SwarmDock runs. Green and orange spheres around the receptor (grey) represent the center of mass of ligand positions before and after enrichment, respectively. (b) For each model of a cluster 109 molecular descriptors are computed and grouped by cluster to quantify the protein–protein interaction. These distributions are characterized by min, Q1, median, Q3 and max that represent the features for a supervised learning algorithm. Finally, a matrix is generated which compares all possible combinations of clusters for each target to train a binary classifier where LRMSD_n_ < LRMSD_m_ produces label 1 otherwise 0. (c) To rank clusters for a new target the classifier is applied to all possible cluster comparisons. Counted is the number of times a cluster was predicted to have a lower LRMSD compared to another cluster. Ranking is based on descending order where the cluster with the highest number is ranked first and the cluster with the lowest number is ranked last.

The method presented here for ranking a set of clusters of docked protein–protein complexes is summarized in Figure 1. This method combines localized enrichment of clusters with additional solutions and training a supervised learning algorithm to distinguish near native from incorrect clusters. The classifier learns from a set of 1092 features of pairwise cluster comparison examples that describing the two clusters. The classifier is optimized to predict whether the LRMSD(cluster_*n*_) < LRMSD(cluster_*m*_). Applying this classifier exhaustively to all possible pairwise combinations of clusters produces a ranking where the best cluster has the highest number of predicted LRMSD(cluster_*n*_) < LRMSD(cluster_*m*_) occurrences and the worst cluster the least number.

**Figure 2:**
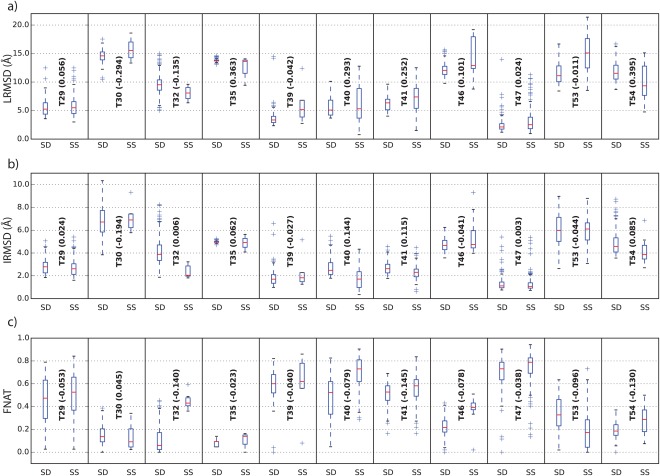
Comparison of score_set (SS) models vs. SwarmDock (SD) local enrichment for (a) LRMSD, (b) IRMSD and (c) FNAT.

### Data set

The machine learning protocol for cluster ranking was trained and tested on previous CAPRI targets as compiled in the score_set dataset. Decoys from the following 13 targets were used: T29, T30, T32, T35, T37, T39, T40, T41, T46, T47, T50, T53, and T54. Targets T36 and T38 were omitted because of the absence of acceptable, medium or high quality models according to the CAPRI assessment standard. The crystal structure of target T40 (PDB: 3E8L) reports two interfaces. We denote the ligand binding position observed in chain C as T40a and for chain B as T40b. Furthermore, targets T37 and T50 were randomly chosen as a hold‐out set to test ranking performance only. Thus, data from these two targets was not used for training. Table 1 provides an overview of the number of models for all targets. This data‐set makes it possible to test the cluster ranking method on a set of decoys from a large variety of different protein–protein docking programs.

**Table 1:** CAPRI‐Targets Overview

						Clusters	Clusters > 5			
Target	Total	High	Medium	Acceptable	Incorrect	Count	Count	Min.	Med.	Max.
T29	2083 (1773)	2 (2)	78 (72)	87 (70)	1916 (1629)	925 (802)	61 (49)	6 (6)	9 (9)	147 (136)
T30	1343 (1106)	0 (0)	0 (0)	2 (2)	1341 (1104)	741 (639)	26 (24)	6 (6)	8.5 (7)	50 (50)
T32	599 (572)	0 (0)	3 (3)	12 (12)	584 (557)	224 (217)	12 (12)	6 (6)	9.5 (9)	168 (166)
T35	499 (467)	0 (0)	0 (0)	3 (2)	496 (465)	198 (193)	14 (12)	4 (3)	8 (7)	131 (128)
T37	1500 (1112)	11 (8)	46 (34)	42 (34)	1401 (1036)	629 (500)	55 (41)	6 (6)	9 (8)	35 (27)
T39	1400 (1261)	0 (0)	3 (3)	1 (1)	1396 (1257)	465 (440)	50 (44)	6 (5)	9 (8)	94 (94)
T40(a/b)	2180 (1886)	193 (176)	206 (163)	189 (149)	1592 (1398)	479 (451)	68 (57)	6 (6)	12 (10)	373 (333)
T41	1200 (1029)	2 (2)	120 (99)	249 (198)	829 (729)	141 (139)	27 (25)	6 (6)	15 (13)	343 (271)
T46	1699 (1321)	0 (0)	0 (0)	24 (24)	1675 (1297)	754 (611)	49 (35)	6 (6)	9 (8)	43 (43)
T47	1051 (988)	278 (278)	307 (301)	26 (20)	440 (389)	84 (82)	24 (20)	6 (6)	15 (10)	607 (595)
T50	1451 (1265)	0 (0)	36 (35)	97 (89)	1318 (1141)	306 (284)	41 (35)	6 (6)	11 (10)	148 (138)
T53	1400 (1191)	0 (0)	17 (9)	113 (92)	1270 (1092)	277 (260)	45 (42)	6 (6)	10.5 (10)	164 (150)
T54	1400 (1215)	0 (0)	1 (1)	18 (18)	1381 (1196)	301 (285)	55 (49)	6 (6)	12 (12)	92 (92)

The total number of clusters and models with high, medium, acceptable and incorrect quality in the score_set dataset are shown. For the cluster‐size cutoff > 5, the minimum, median and maximum number of models in a cluster are shown. Numbers in brackets indicate the number of models or clusters after removing solutions with steric clashes.

Models with steric‐clashes as assigned in the score_set dataset were removed, missing side‐chain atoms were modeled with SCWRL^25^ and receptor/ligand chains were truncated to remove residues not shared by all models in a target.

### Model assessment measures

The model quality is quantified by computing the fraction of native contacts (FNAT), interface root mean square deviation (IRMSD) and the carbon‐alpha (Cα) ligand root mean square deviation (LRMSD). For details of the computation of the FNAT and IRMSD please see Refs. 26, 27. The LRMSD is computed by superimposing the receptor chains to their equivalent Cα atoms, followed by an LRMSD calculation based on the ligand Cα atoms. This LRMSD calculation differs from the standard CAPRI procedure where backbone atoms are used for both super‐imposition and the calculation of backbone deviation. The model quality (i.e., incorrect, acceptable, medium or high) annotations are taken from the assignments in the score_set dataset and are based on backbone LRMSD, IRMSD, and FNAT as described in reference.^22^

### Clustering

The models for each target were clustered with the GROMOS^28^ clustering algorithm with a 10 Å LRMSD cutoff which is implemented in the GROMACS software package.^29^ This produces clusters where all members of a cluster are within 10 Å from the centroid. Table 1 gives an overview of the number of clusters per target. Initially, two other clustering algorithms were tested, namely single‐linkage^30^ and Jarvis‐Patrick clustering.^31^ These two clustering algorithms produced unfavorable extended ligand‐clusters that are spread over large areas of the receptor surface for some targets. Hence, these two methods were rejected as viable alternatives to GROMOS.

The aim of this work is to reliably locate true‐positive binding regions. To this end we imposed a cutoff on the size of clusters to contain > 5 solutions since, in general, if the docking community is likely to find the true‐positive region it is likely to be populated by more than one or just a few solutions. However, for the two targets T35 and T39 the cluster size for near‐native solutions was 3 and 5, respectively. To be still able to perform a ranking of the near‐native cluster, and have more data for training/testing/cross‐validation, these were included into the set regardless of our initial cutoff.

### Cluster enrichment

The number of models contained in a cluster can vary significantly. To balance this information deficit, localized SwarmDock runs, to generate additional solutions for each cluster and populate the binding funnel, were performed. A total of 250 particles were optimized in the swarming for each cluster, with starting positions for each particle being within the 10 Å LRMSD limit of each cluster. The ligand‐receptor starting conformation selected for swarming was taken from the unbound state of each target. To encourage additional sampling within the environment of each cluster SwarmDock was not allowed to fully converge. Therefore, the final ensemble of SwarmDock derived docking poses for each cluster was typically <10 Å but not under 3 Å. To minimize the occurrence of clashes, structures generated with SwarmDock were energy‐minimized employing the steepest decent algorithm implemented in CHARMM.^32^ If structures with steric clashes between the receptor‐ligand atoms are still present after this step, they were removed from the set. Where a steric clash is defined as two atoms overlapping by their van der Waals radii. This clash criterion is more stringent than the CAPRI definition for clashes where a clash between two heavy atoms is defined by a distance below 3 Å.

This methodology not only allows a higher density sampling within the bounds of each cluster but also enables a deeper descent of the true binding funnel, should a cluster represent such a funnel.

To make the computation of molecular descriptors tractable the additional solutions for each swarmed cluster were sub‐clustered again, using a 3 Å cutoff, with the GROMOS algorithm. These sub‐clusters were ranked according to size. For each of the top ten clusters the model closest to the sub‐cluster centroid was taken. This resulted in 10 additional models, if 10 or more clusters were present. This enrichment data is used for the subsequent computation of molecular descriptors with the aim to gain a better estimate of the local energy but are not explicitly used as model poses that may be improvements over the original score_set models.

### Computation of molecular descriptors and feature construction

A total of 109 molecular descriptors from the CCharPPI server^33^ were computed for each model from the CAPRI decoy set and the cluster enrichment. These include residue contact and distance dependent potentials (rc), atomic contact and distance dependent potentials (ac), constituent terms of statistical potentials (sp), composite scoring functions (cs), solvation energy functions (se), and van der Waals and electrostatic potentials (ve). Table 2 provides an overview of the number of descriptors for each category. A detailed list of all molecular descriptors is provided online on the CCharPPI server website (https://life.bsc.es/pid/ccharppi/info/help_descriptors).

**Table 2:** Categories of Molecular Descriptors Used in this Work

Category	Abbreviation	Nb. Molecular Descriptors	Description
Residue contact and distance potential	rc	34	Corse‐grained residue potentials between intermolecular residues.
Atomic contact and distance potential	ac	21	Fine grained atomic potential between intermolecular atoms.
Statistical potential constitute terms	sp	18	Knowledge based potential terms from^34^
Composite scoring functions	cs	11	Scoring functions composed of different weighted additive terms.
Solvation energy functions	se	5	Functions describing the effect of desolvation upon protein–protein complex formation.
Hydrogen bonding	hb	3	Intermolecular hydrogen bonding
Van der Waals and electrostatic	ve	6	Contribution of intermolecular van der Waals and electrostatics forces such as attractive and repulsive terms.
Miscellaneous	mi	11	Functions describing amino acid propensity, interface packing and change in rotational and translational entropy

Descriptors are standardized by scaling the data points to zero mean and unit variance for each complex. Finally, the values are aggregated by cluster, providing 109 distributions [see Fig. 1(b)]. The cluster distributions are characterized by five points: minimum (MIN), 1st quartile (Q1), median (AVG), 3rd quartile (Q3), and maximum (MAX). In addition to these descriptors the cluster size was added as a feature. This results in 546 features describing a cluster. As an example, the feature with the name C2_Q1_N_CP_TB denotes a feature calculated for the second cluster (C2) in a comparison and represents the 1st quartile (Q1) of a normalized distribution (N) for the TOBI potential (CP_TB).

### Training, testing and ranking

A pairwise cluster comparison matrix is constructed which compares all possible binary cluster combinations for each target where every comparison occurs only once in the matrix. Each row contains the 546 features of a cluster m and n resulting in 1092 features in total per training example [see Fig. 1(b)]. This matrix is used to train an extremely randomized tree classifier (ERT) from the scikit‐learn machine learning library^35^ to assign the label 1 if min(LRMSD_n_) < min(LRMSD_m_) otherwise 0.

The ERT classifier was trained with 3000 trees where samples are bootstrapped and the gini impurity criterion is used to decide on the quality of splits when building trees. Out‐of‐bag samples were used to estimate the generalization error. Individual trees use $\sqrt{1092}$ features (33 features) at once and have a maximum depth of 100 where the minimum sample size per leave is 1. These numbers represent empirical good values for classification tasks for tree‐based classifiers.^36^, ^37^

Leave‐Complex‐Out Cross‐Validation (LCO‐CV) was used to test the performance of the classifier. Where every fold uses the data for *n* − 1 targets for training and leaves out all training examples for the target it is being tested on, yielding 11 cross‐validations.

Employing the above classifier, cluster ranking is based on the number of times a cluster was predicted to have a lower LRMSD than another, where the cluster with the highest number is ranked first and the cluster with the least assignments is ranked last [see Fig. 1(c)]. The ranking performance is benchmarked by its ability to rank the cluster that contains the model with the lowest LRMSD solution from the score_set model. We refer to these clusters as lowest LRMSD cluster or best near native cluster. The results are compared to a base‐line ranking protocol where the same clusters containing score_set and enrichment models are ranked with a DIFRE based potential for protein–protein complexes known as DCOMPLEX^38^; here clusters are ranked according to the model with the lowest energy within each cluster. The DCOMPLEX function is a statistical potential optimized to produce low energies for single near‐native models and was not explicitly trained to rank clusters.

### Molecular descriptor, feature and classifier performance measures

The importance of the molecular descriptors with respect to their power to discriminate between near‐native and incorrect clusters was assessed using the Mann‐Whitney *U* test. In addition, the correlation of two molecular descriptors is calculated with the Pearson's Product Momentum Correlation Coefficient (PPMCC).

The relative importance of features for classification is computed by the internal ERT feature importance function, the expected fraction of samples upon which a feature will have bearing, a function of feature occurrence in trees and its position in the tree.

The classification performance of the ERT classifiers is measured by the following metrics:

Recall: TPTP+FN

$$\mathrm{Pr}\text{ecision}:\;\frac{\text{TP}}{\text{TP}\;+\;\text{FP}}$$

$$\text{F1}‐\text{Score}:\;\frac{2\times \text{precision}\times \text{recall}}{\text{precision}\times \text{recall}}$$

$$\text{Accuracy}:\;\frac{\text{TP}+\text{TN}}{\text{TP}+\text{FP}+\text{FN}+\text{TN}}$$

### Feature space reduction and transformation

The dimensionality of the feature space was reduced with three different methods to test its effect on the performance of the classifier, namely: factor analysis (FA), PCA, and kernel PCA with a radial basis function (KPCA). The dimensionality of the feature‐space was incrementally reduced from 1092 dimensions (maximum number of features) to 2 in steps of 10 dimensions. For each step the transformer was fitted using training data, the fitted model was then applied to transform the test data. The selection of training and test split was performed with LCO‐CV resulting in 11 splits. The ERT classifier was trained with the transformed data of each split and dimension and performance metrics such as recall, precision, F1‐score and accuracy were calculated.

The ranking performance of the ERT classifier that has the best average F1‐score from the LCO‐CV was tested. Thus, three models are generated: ERT + PCA (i.e., ERT classifier trained and tested on transformed data with PCA), ERT + FA (i.e., ERT classifier trained and tested on transformed data with FA) and ERT + KPCA (i.e., ERT classifier trained and tested on transformed data with KPCA).

### Recursive feature elimination

The goal of the recursive feature elimination (RFE) is to reduce the initial number of features (1092) by recursively removing the least important features. Initially, the ERT classifier is trained on the full set of features and weights are assigned to each one of them according to their relative feature importance computed from the ERT classifier. Features whose absolute weights are smallest are then removed from the current set. Feature pruning is done in increments of 10 features in each round and the weights are based on average feature importance from each target of the LCO‐CV. The ERT performance for each subset of features is assessed based on average accuracy, precision, recall and F1 scores. The ERT classifier with the best F1 performance is selected for benchmarking the ranking performance and denoted as ERT + RFE in the following sections. Additionally, feature space transformation with FA was tested again on the reduced set and we refer to this model as ERT + RFE + FA.

## RESULTS AND DISCUSSION

The aim of this work is to establish a machine learning protocol which is able to rank near native clusters of docked protein–protein complexes over incorrect ones by using a wide set of currently available molecular descriptors important for protein–protein interactions. The method makes use of localized re‐sampling of clusters [see Fig. 1(a)], uses features based on the distribution of molecular descriptor values and uses a pairwise cluster comparison representation to learn how to distinguish near native from incorrect clusters [Fig. 1(b,c)].

In summary, the best classifier based on ERT + RFE was able to rank near‐native clusters in the top 10 in all targets if such a cluster was present with the cluster‐size cutoff > 5. However, for targets T35 and T39 this near native cluster was excluded. For 5 targets the near‐native cluster was ranked in the top 1 and for 9 targets the best cluster was ranked in the top 5. Table 3 provides a summary of each individual rank for the lowest LRMSD near native clusters for all targets.

**Table 3:** Model Performance for Each Target

Target	Best NN Cluster, Nb. of Models (H/M/A/I)	Best NN Cluster, best Model (Å)	Best NN Cluster, centroid Model (Å)	DC	ERT	ERT+FA	ERT+PCA	ERT+KPCA	ERT+RFE	ERT+RFE+FA
T29	2/72/60/2	3.01 (H)	3.80 (M)	7	1	1	1	1	1	1
T30	0/0/2/7	13.32 (A)	13.32 (A)	12	8	7	7	8	8	6
T32	0/3/6/0	6.35 (M)	6.56 (M)	10	9	8	5	6	9	5
T35	0/0/2/1	9.44 (A)	9.44 (A)	12	9	12	12	12	10	12
*T37	1/14/8/0	3.61 (H)	8.30 (M)	1	3	15	10	4	4	2
T39	0/3/1/1	2.75 (M)	2.75 (M)	38	12	13	35	39	4	33
T40a	90/139/104/0	0.76 (H)	4.21 (M)	1	5	1	1	1	6	3
T40b	86/20/11/15	0.57 (H)	1.22 (H)	2	2	25	3	2	1	2
T41	2/99/170/0	1.50 (H)	5.41 (M)	3	1	1	1	1	1	1
T46	0/0/12/25	8.77 (A)	12.83 (I)	1	3	2	3	3	2	1
T47	278/301/14/2	0.96 (H)	1.38 (H)	4	1	1	1	1	1	1
*T50	0/35/85/18	2.22 (M)	6.74 (M)	1	1	1	1	1	1	1
T53	0/9/66/75	9.44 (M)	15.11 (A)	2	2	1	1	1	1	1
T54	0/1/7/8	4.76 (M)	8.72 (A)	1	5	5	5	6	3	3
Top 1				5 (4)	4 (4)	6 (5)	6 (5)	6 (5)	5 (6)	6 (6)
Top 5				8 (8)	9 (9)	8 (8)	9 (9)	8 (8)	9 (10)	10 (10)
Top 10				10 (10)	12 (12)	10 (9)	10 (10)	11 (11)	13 (13)	11 (11)
Avg. Rank				7.2 (7.2)	4.6 (4.4)	5.2 (7.1)	6.4 (6.5)	6.5 (6.5)	3.9 (3.5)	5.4 (5.3)
Rel. Imp.					35% (39%)	27% (2%)	11% (10%)	10% (10%)	45% (51%)	25% (27%)

The rank of the cluster that contained the model with the lowest LRMSD to the crystal structures are shown, referred to as best near native (NN) cluster. Number of models for this cluster with high (H), medium (M), acceptable (A) and incorrect (I) are shown along the LRMSD of the best and centroid model in the cluster. The summary Top 1, Top 5, Top 10 shows the number of times a cluster was ranked in the respective top n category out of all 13 targets. The rows Avg. Rank and Rel. Imp. report the average rank and its relative improvement to DCOMPLEX (DC) respectively. The summary rows Top 1, Top 5, Top 10, Avg. Rank, Rel. Imp. first report values considering interface T40a and in brackets for interface T40b. The * indicates targets used in the hold‐out set.

In this section the method and training strategy and its implications are discussed. An analysis is presented on how enrichment of clusters with localized SwarmDock affects the accuracy of LRMSD, IRMSD and FNAT. Finally, a discussion of the performance of the different molecular descriptors, how feature space transformation with FA, PCA and KPCA is affecting the prediction accuracy, and how feature elimination can help to build a simpler model without losing accuracy, is presented.

### Ranking with statistical learning

The motivation behind cluster based ranking instead of focusing on single model ranking is founded in the observation that protein–protein complex formation is initiated by so called encounter complexes that differ in conformation, rotation and translation from the final docked pose observed in the crystal structure but play an important role in the recognition process. Furthermore, this is supported by the good performance of ensemble and cluster‐based scoring schemes in previous CAPRI rounds.^39^, ^40^ Ranking protocols that made use of minimum cluster energies from a scoring function such as DCOMPLEX (that is, used in SwarmDock) usually performed better than non‐cluster based approaches.

Furthermore, with the method presented here a solution is provided which addresses the problems of cluster‐size imbalance and class‐bias of training examples that is a marked problem for many machine‐learning problems.^41^

The clustering of docked protein–protein complexes by LRMSD results in a power law distribution of cluster size where only a few clusters have large number of models and most clusters have few models (see Supporting Information Figs. S3–S15). This underrepresentation of solutions for many clusters can be a problem when conclusions are derived from distribution points such as median, first quartile, third quartile, minimum and maximum. To address the problem of cluster size imbalance, and hence an information bias, a localized enrichment of clusters with SwarmDock is performed. By applying enrichment to clusters additional ligand models with translational, rotational and conformational variance are created thus resulting in more accurate estimates of median, first quartile and third quartile across the minima in the local energy landscape.

The second problem of class bias occurs when one class is proportionally under‐represented compared to another during supervised learning. A classifier trained on a class‐biased set of examples and optimized for accuracy will result in a classifier with poor sensitivity toward the under‐represented class. This class‐bias is the case for decoy sets of protein–protein complexes, where the number of models for the two classes of near‐native (i.e., acceptable, medium and high quality models) and incorrect solutions has a large difference. In the score_set dataset used here, only 11% of the decoys have near native solutions. Hence, a training approach where a classifier is trained based on an assignment of near native or incorrect labels would have this class bias problem.

Instead the training strategy presented here focuses on learning from pairwise cluster comparisons [see Fig. 1(b)]. In this approach every possible combination of two clusters n and m are compared based on 1092 features (i.e., 546 features describing each cluster) for each comparison. The supervised learning task is now to predict which of the two clusters has the lower LRMSD (i.e., two class prediction). This strategy has three advantages, namely i) this allows the training of a classifier from a limited number of protein–protein complexes; constructing a cluster comparison matrix enables the gain of 7248 training examples (i.e., the number of all possible pairwise comparisons), ii) the class bias problem is resolved which initially appeared from a small fraction of near native solutions in the decoy set and iii) this method not only provides a ranking of clusters but implicitly learns to rank according to LRMSD too.

### Clustering and the effect of localized enrichment on near native clusters

The clustering produces clusters with up to 10 Å LRMSD difference to the centroid where the best near native cluster in the test set is highly populated with solutions of acceptable or better quality with only few incorrect models (see Table 3). In general, the localized enrichment with SwarmDock produced additional solutions within a limited range of values for LRMSD, IRMSD and FNAT [see Fig. 2].

This method has a modest success in improving the quality of the models in near native clusters. The LRMSD improved for 4 out of 11 targets (T30, T32, T39, T53). The largest improvement was observed for T30 where the LRMSD dropped by 2.95 Å. Similar improvements are seen for IRMSD, here again 4 out of 11 target improved (T30, T39, T46, T53) where the largest improvement was observed for target T30 with an improvement of 1.94 Å. Finally, the FNAT was only marginally improved for target T30 by 0.045.

As the results indicate, improving the quality of near native‐clusters is a hard problem. One reason is the already good quality of models in near‐native clusters within score_set: a total of 4 out of the 11 targets have models with high quality (T29, T40, T41, T47) and a further 4 targets have near‐native clusters with medium quality models (T32, T39, T53, T54). Only 3 targets, namely T30, T35, and T46 have near‐native clusters with only acceptable models.

These results suggest that the normal modes generated for receptor‐ligand conformations with SwarmDock are often not able to find the conformational transitions necessary to improve LRMSD, IRMSD or FNAT for this particular test set.

### Molecular descriptors, discriminative power and cross‐correlation

**Figure 3:**
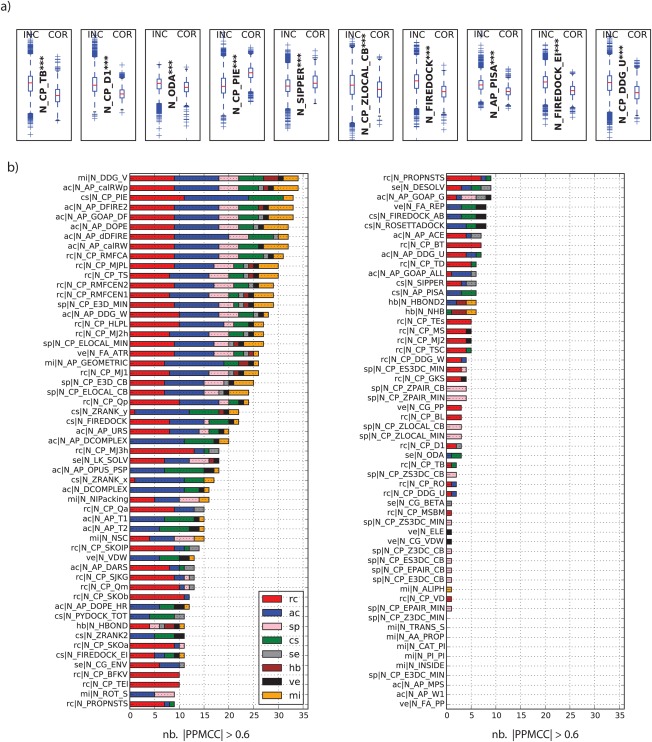
(a) The top 10 molecular descriptors comparing the distribution of values for near‐native clusters (COR) versus clusters containing only incorrect solutions (INC). Stars indicate *P* values for *U* test between groups COR and INC (***: *P* values < 0.0001, **: *P* values < 0.001 and *: *P* values <0.01). (b) Number of highly correlated molecular descriptors with a |PPMCC| > 0.6 colored by category.

The analysis of all 109 molecular descriptors with respect to their power to distinguish between near‐native and incorrect clusters, based on *P*‐values from a *U* test shows that 99 out of 109 molecular descriptors are able to produce significant difference with a *P* values < 0.01. Figure 3a shows the distribution of values for the top 10 molecular descriptors with a *P* values < 0.0001 (all other molecular descriptors are shown in Supporting Information Fig. S1). Although 99 have a significant difference between the two groups near‐native and incorrect, their value range heavily overlaps. The best descriptor N_CP_TB (TOBI potential^42^), for example, has good discrimination at the 25% to 75% level (first quartile to third quartile); however, the high number of low energy outliers in the incorrect clusters make a clear separation of the two groups hard.

This is a common theme for all descriptors where the high number of outliers makes it impossible to get a reliable separation of near‐native and incorrect clusters.

To test the unique information value of a molecular descriptor the PPMCC was calculated for all possible pairs. A PPMCC > 0.6 indicates a strong positive correlation and a PPMCC < −0.6 indicates a strong negative correlation of two pairs.

The heat‐map in Supporting Information Fig. S2 shows correlations between all possible pairs and the number of highly correlated molecular descriptors [|PPMCC| > 0.6; see Fig. 3(b)]. The data shows that 11 descriptors have a high correlation with > 30 other descriptors. The two highest correlated descriptors, N_DDG_V and N_AP_calRWp is a microscopic surface energy model^43^ and the calRWplus orientation‐dependent atomic potential described in Ref. 44. The other descriptors in the top 11 are either reside‐contact/distance potentials or atomic‐contact/distance potentials and have a high correlation to descriptors in both mentioned categories. The exception here is N_CP_PIE (PIE score^45^). Interestingly, The TOBI potential (N_CP_TB), one of the highest discriminative descriptors has only high correlation with two other descriptors (N_CP_PIE and N_CP_TSC^46^), hence, has high non‐redundant information value. Low correlation is also observed for the 10 descriptors with no statistically significant discriminative power between near‐native and incorrect clusters [see Fig. 3(b)].

### Ranking and feature performance of the ERT classifier

**Figure 4:**
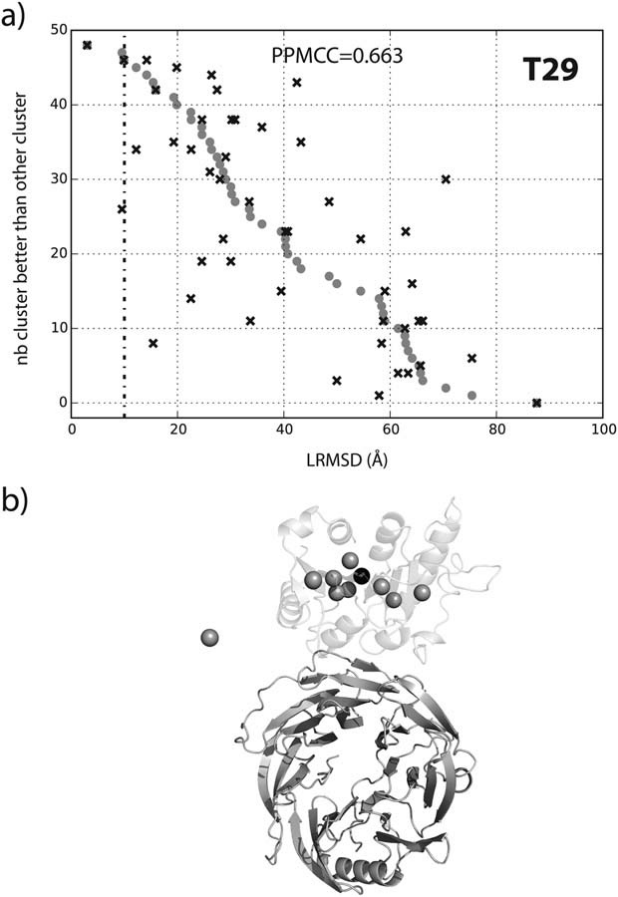
Predictions for T29 based on the standard ERT classifier. (a) the predicted number of times a cluster is better vs. all other clusters (black cross) compared to the actual values (gray dots). The LRMSD values on the x axis are based on the cluster member with the lowest LRMSD. The bottom panel (b) shows the receptor (dark gray cartoon representation) and a sphere indicating the center of mass of the centroid model for each cluster. The top 10 ranked clusters (black: rank 1, gray: rank 2–10) are shown. The transparent cartoon indicates the observed position of the ligand from the crystal structure (PDB: 2VDU^47^).

First, the performance of the ERT classifier with all 1092 features was tested for each individual target from score_set (see Table 3). In summary, this method is able to rank the lowest LRMSD near‐native clusters in the top 1 for 4 targets, in the top 5 for 9 targets and the top 10 for 12 targets with an average rank of 4.6 (4.4 for T40b) with a relative improvement of 35% (39% for T40b) compared to DCOMPLEX. Figure 4a shows the cluster ranking for T29 where a good correlation coefficient of 0.663 compared to the actual values is achieved. Here it can be seen that the best cluster with a minimum LRMSD of 3.0 Å was ranked first. Furthermore, 9 out of the top 10 ranked clusters are in close proximity to the best near‐native cluster [see Fig. 4(b), information for other targets can be found in Supporting Information Fig. S16].

**Figure 5:**
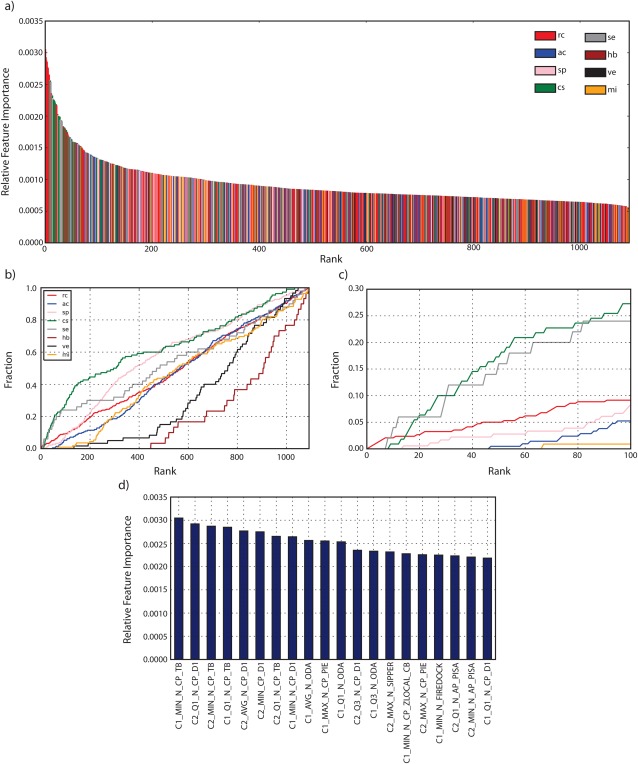
Feature importance. The top panel (a) shows the relative importance of all 1092 features colored by category. The bottom panels show the cumulative fraction of use of features for different categories for feature ranks 1 to 1092 (b) and 1 to 100 (c). Panel (d) shows the relative feature importance of the top 20 features.

An analysis of the feature importance for the ERT classifier shows a drop of the relative importance after the first 20 features [see Fig. 5(a)], which is an expected behavior of a random‐tree based classifier.^48^ However, no feature is dominant with respect to their relative importance and ranges from 0.0030 to 0.0007 for all 1092 features used. Furthermore, the most important molecular descriptor categories are rc, cs, and se, where the top eight features alone are rc.

Comparing the fraction of features used versus rank [see Fig. 5(b,c)] a clear pattern emerges where features from the categories cs, se, rc, and sp have a high dominance. Values of 42% for cs, 30% for se, 21% for sp and 20% for rc when counting the fraction of features used from rank 1 to rank 200 for each category are observed. The plot in Figure 5(b,c) also shows that features of the category hb and ve are consistently under‐employed and fall behind the other 6 categories by a large margin. Features from these two categories are first observed at rank 420 and 150, respectively.

A detailed look at the top 20 features [see Fig. 5(d)] shows that the minimum TOBI potential (rc) of the first cluster in a pairwise‐comparison (C1_MIN_N_CP_TB) is ranked first. Additionally, the TOBI potential based features C2_MIN_N_CP_TB, C1_Q1_N_CP_TB and C2_Q1_N_CP_TB rank 3,4, and 7, respectively. Another molecular descriptor with high occurrence in the top 20 is the DECK residue level distance‐dependent potential^49^ (N_CP_D1, rc). Features based on this descriptor have 6 occurrences in the top 20 namely C2_Q1_N_CP_D1 (rank 2), C2_AVG_N_CP_D1 (rank 5), C2_MIN_N_CP_D1 (rank 6), C1_MIN_N_CP_D1 (rank 8), C2_Q3_N_CP_D1 (rank 12), C1_Q1_N_CP_D1 (rank 20). Other molecular descriptors in the top 20 are optimal docking area (N_ODA, se),^34^ PIE score (N_CP_PIE, cs), SIPPER (N_SIPPER, cs),^50^ w_local Z‐score C_beta potential (N_CP_ZLOCAL_CB, sp),^51^ FireDock energy (N_FIREDOCK, cs)^52^ and the PISA score (N_AP_PISA, cs).^53^

This data suggests that coarse grain potentials based on residue contacts such as the TOBI potential or the DECK scoring function are better at distinguishing near‐native from incorrect clusters compared to atomic potentials such as DCOMPLEX.

Furthermore, functions describing the contribution of hydrogen bonding or VdW/electrostatic forces have a very low predictive power and consistently underperform [see Fig. 5(b)]. This can be explained by the intrinsic heterogeneity of the score_set data, which originates from a large number of different protein–protein docking algorithms. Many of these are rigid‐body (e.g., ZDOCK^54^ or PIPER^55^) and are not able to include conformational transitions from unbound to bound. Furthermore, large numbers of structures in the score_set are not locally optimized by energy‐minimization or other refinement methods, which would optimize for hydrogen bonding and VdW/electrostatic thus making it hard to estimate correct values for definitive identification of near‐native and incorrect clusters.

### The effect of feature space transformation on prediction accuracy

**Figure 6:**
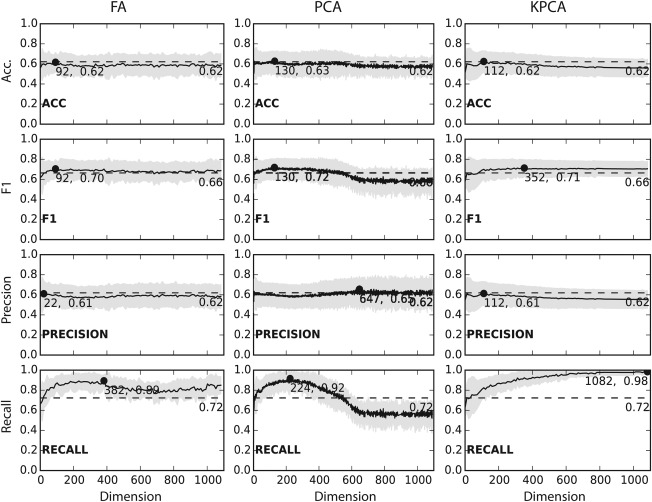
Comparison of FA, PCA, KPCA. Shows the change of accuracy, F1, precision and recall for feature space transformations with FA, PCA and KPCA with a rbf kernel. The solid black line shows the mean value and the light gray area indicates the standard deviation. The dotted gray line indicates the performance of the classifier without applied feature space transformation. Spheres indicate the best dimension with the highest value.

The application of dimensionality reduction has a marked, positive impact on the recall of our classifier. The results in Figure 6 show a maximum increase from 0.72 for the standard ERT classifier to 0.89 (382 dimensions), 0.92 (224 dimensions) and 0.98 (1082 dimensions) for FA, PCA, and KPCA, respectively.

However, results for accuracy, precision and F1 indicate more moderate changes. For FA and KPCA the average accuracy calculated from all LCO‐CV stays at 0.62 (dimensions 92 and 112, respectively) and goes slightly up to 0.63 (dimension 130) for PCA. Similar results were observed for precision where the values decrease slightly to 0.61 for FA and KPCA (dimensions 22 and 112) and increases slightly to 0.65 (dimension 647) from a base value of 0.62. The F1 increases for all three methods almost equally from 0.66 to 0.70 (dimension 92), 0.72 (dimension 130) and 0.71 (dimension 352) for FA, PCA, and KPCA. This increase in F1 is mainly due to the stark increase in recall.

The ERT classifier, with feature space transformation has a positive effect on the top 1 ranking performance in the benchmark set (see Table 3). The relative success to rank the lowest LRMSD near‐native cluster within the top 1 increases from 31% to 46% for FA, PCA, and KPCA. When the second interface T40b is considered this improvement drops to a 38% success rate. However, the performance in the top 5 from 69% stays unchanged for PCA and drops to 62% for FA and KPCA. Similar effect on ranking is observed for the top 10, where the success rate drops from 92% to 77% for ERT + FA and ERT + PCA and to 85% for ERT + KPCA. Overall, the average rank for T40a increases from 4.6 to 5.2, 6.4 and 6.5 for ERT + FA, ERT + PCA and ERT + KPCA, respectively. Including the second interface, T40b, an average rank of 6.5 is observed for models ERT + PCA and ERT + KPCA. However, for model ERT + FA the rank for this second interface of T40 is 25 which results in an increase in the average rank from 5.2 (T40a) to 7.1 (T40b).

The drop in overall ranking ability for ERT + PCA and ERT + KPCA can be attributed to the decrease in ranking performance for target T35 and T39. For target T35 the rank for the lowest LRMSD near‐native cluster drops from 9 to 12 for all three transformations. This change is even more marked for T39 where the initial rank drops from 12 to 13 (ERT + FA), 35 (ERT + PCA) and 39 (ERT + KPCA). This decreased ranking performance for targets T35, T39, and T40b results in only a modest relative improvement compared to DCOMPLEX. Where the models improve by 27% (ERT + FA), 11% (ERT + PCA) and 10% (ERT + KPCA) considering T40a. This value drops even further when T40b is considered where the improvement now resembles 2% (ERT + FA), 10% (ERT + PCA) and 10% (ERT + KPCA).

### Optimization of the number of molecular descriptors

Supporting Information Figure S1 shows that not all molecular descriptors have a significant predictive power to distinguish between near‐native and incorrect clusters with a *P* values < 0.01 and that a large number of descriptors have high co‐linearity (see Supporting Information Fig. S2). And indeed a recursive feature elimination procedure shows that the number of features could be decreased from 1092 to 402 and even improves the top 1 and top 10 and average ranking performance. In essence an ERT classifier trained on the reduced set of features, referred to as ERT + RFE, produces a 100% success rate to rank the best near‐native cluster within the top 10 and has an average rank of 3.9 or 3.5 considering T40a or T40b, respectively, down from 4.6 (T40a) or 4.4 (T40b) compared to an ERT classifier trained on all features.

**Figure 7:**
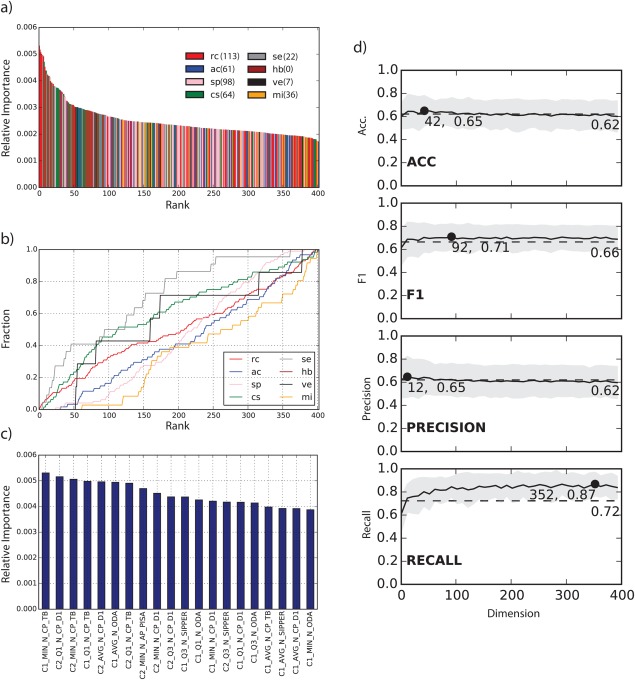
Analysis of the reduced feature set after RFE. (a) the relative feature importance for all 402 features colored by descriptor category. (b) fraction of features used from one of the 8 categories versus rank. (c) relative importance of the top 20 features. (d) change of accuracy, F1, precision and recall of the ERT + RFE + FA classifier for different dimensions.

All features based on hydrogen bonding are dropped and only seven features describing intermolecular electrostatic/van der Waals forces remain. In total the reduced set consists of features from the categories rc (113), ac (61), sp (98), cs (64), se (22), hb (0), ve (7) and mi (36). The distribution of the relative feature importance on the reduced set is similar to the full set [see Figs. 4(a) and 7(a)]. Here again features of categories rc, se, and cs compromise most of the top 50 ranks; the fraction of features used versus the rank [Fig. 7(b)] makes this clear, where these three categories make up a high fraction early on. Inspection of the top 20 features [Fig. 7(c)] reveals that features based on the TOBI potential occur 6 times with the best feature being C1_MIN_N_CP_TB. As in the full feature set, features based on DECK (7), ODA (4), PISA (1) and SIPPER (3) are present in the reduced set too.

Dimensionality reduction with FA produced only significant improvements for the recall, which had a maximum increase at 352 dimensions to 0.87 from 0.72.

For accuracy, F1 and precision the value changed from 0.62 to 0.65 (42 dimensions), 0.66 to 0.71 (92 dimensions) and 0.62 to 0.65 (12 dimensions), respectively.

Rankings based on the ERT classifier with a reduced feature set and FA with 92 dimensions (ERT + RFE + FA) improved the top 1 and top 5‐success rate slightly from 38% to 46% and 69% to 77%, respectively. However, the top 10 success rate decreased from 100% to 85%. This change is due to lost accuracy for targets T35 and T39 where the best near‐native cluster was ranked 12 (10) and 33 (4), respectively (see Table 3). Rankings for all other targets improved or remained unchanged.

## CONCLUSIONS

We have developed a machine learning protocol to distinguish near native from incorrect docked pose clusters. This protocol is based on a set of 1092 features describing the cluster distribution of 109 molecular descriptors. The ERT classifier learns from a set of 7248 pairwise‐cluster comparisons generated from 11 CAPRI‐targets. Localized SwarmDock enrichment was employed to overcome the problem of the power law distribution of clusters, where only a few clusters have a large number of models and a large number of clusters have few models.

The machine learning protocol was benchmarked in a LCO‐CV fashion and used two targets as hold‐outs (i.e., T37 and T50). For our best performing model (ERT + RFE) the average rank of the best near‐native cluster is 3.5 and represents a 51% improvement against a benchmark using DCOMPLEX, a statistical potential for single model protein–protein complexes, to produce a ranking according to the model with the lowest energy within each cluster. However, a user of this methodology has to be aware of the potential limitations of using a cluster size cutoff. The current cutoff used in this work, > 5, did exclude true‐positive solutions for targets T35 and T39 and would decrease the 100% success rate to 85% to rank the best near native cluster within the top 10. Unfortunately, using a lower cutoff results in increased computational overhead for the enrichment and feature calculation and could possibly affect recall and precision. Subsequent work will focus on the property of this parameter.

Furthermore, the analysis of the molecular descriptor set has shown that 99 out of the 109 descriptors are able to distinguish the two groups of near‐native and incorrect clusters with a *P* values of 0.01 or lower. However, outliers in both groups make a clear separation difficult. Furthermore, a large set of molecular descriptors possesses high co‐linearity (that is, |PPMCC| > 0.6) where correlations with up to 39 other descriptors from all categories occurred. Even though tree based classifiers such as the used ERT are robust against co‐linearity a dimensionality reduction with FA, PCA, KPCA or RFE can be helpful. Indeed, the results show partial success, with improved recall, top 1 and top 5 success rates for FA, PCA and KPA. However, we experience a negative effect for targets T35 and T39 where the predicted rank decreases. This is especially stark for T39 where the rank can drop from 4 (ERT + RFE) to 33 (ERT + RFE + FA).

Finally, features based on coarse‐grained descriptors considering residue–residue contacts have been shown to be better at distinguishing near‐native from incorrect clusters compared to features based on atomic contacts, hydrogen‐bonding, electrostatics or van der Waals forces. The latter features are likely to be more effective in scoring poses nearer to the bottom of the native binding funnel, poses that are rarely sampled by current docking methods.

In the future we plan to make our cluster ranking approach available as an option within the SwarmDock webserver thereby enabling identification of potential native docking regions for further refinement.

## Supporting information

Supporting Information.
