# Supplementary material for: A machine learning approach for ranking clusters of docked protein‐protein complexes by pairwise cluster comparison
Source: Proteins. 2017 Jan 20;85(3):528–43. doi: 10.1002/prot.25218 (PMC5396268; doi:10.1002/prot.25218)
Supplement: Supplementary file 1 — Supporting Information. [file PROT-85-528-s001.docx]

**SUPPLEMENTARY MATERIAL**

**Figure S1:** Molecular descriptors comparing the distribution of values for near native clusters (COR) versus clusters containing only incorrect solutions (INC). Stars indicate p-value for U-test between groups COR and INC (***: p-value < 0.0001, **: p-value < 0.001 and *: p-value <0.01).


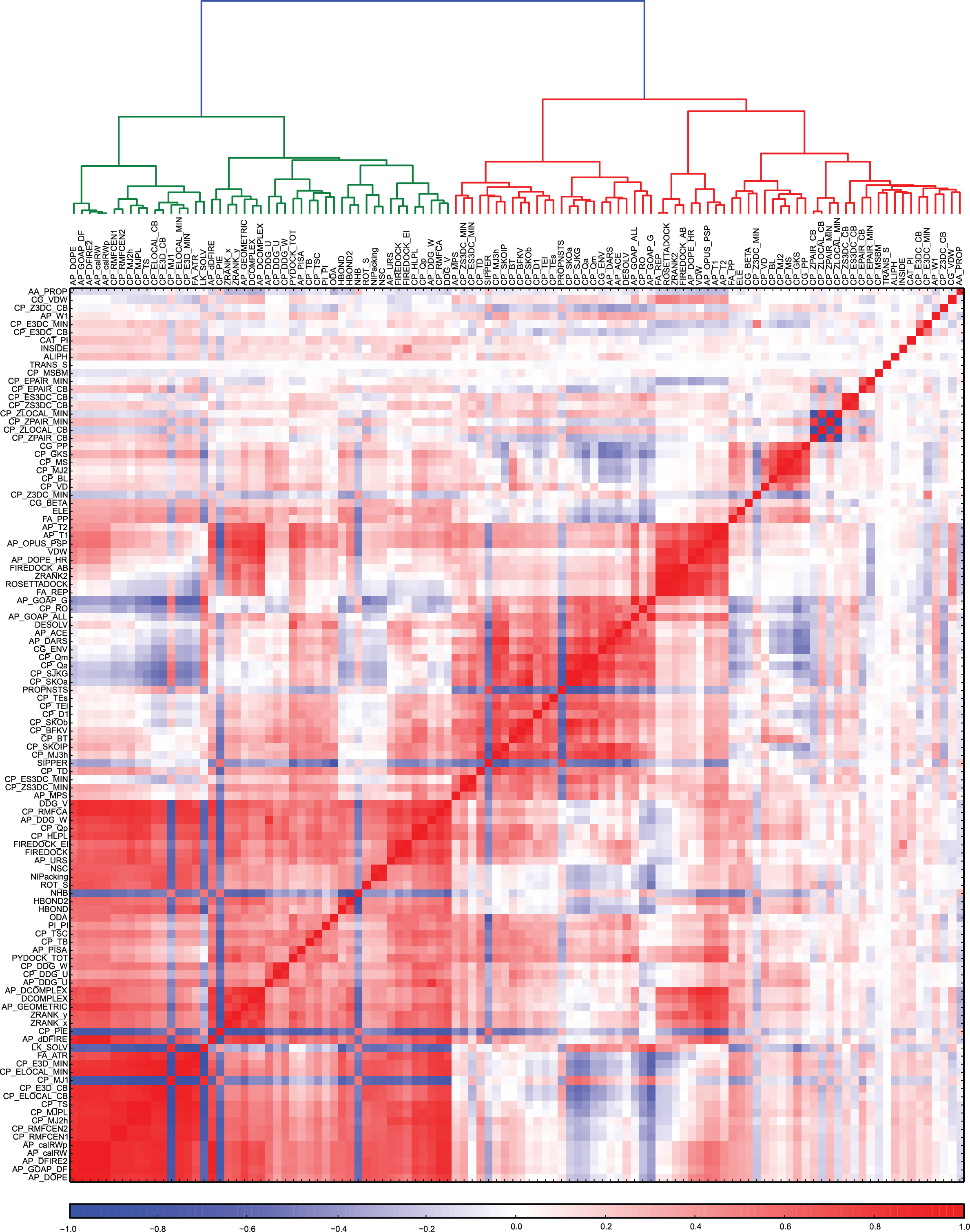


**Figure S2:** Co-linearity of all molecular descriptors. The heat-map shows the PPMCC of all pairs of molecular descriptors. Red and blue indicate high positive and negative correlation respectively. White indicates no correlation. The grouping of the molecular descriptors is based on hierarchical clustering where the distance d = sqrt(2(1-|r|)).

**Figure S3:** T29; CA-LRMSD distribution of all clusters used for testing and training. Labels on the y-axis indicate the number of high (H), medium (M), acceptable (A) and incorrect (I) solutions per cluster. The colored sphere (green: high; orange: medium; red: acceptable; black: incorrect) in each boxplot indicates the CA-LRMSD of the cluster centroid.

**Figure S4:** T30; CA-LRMSD distribution of all clusters used for testing and training. Labels on the y-axis indicate the number of high (H), medium (M), acceptable (A) and incorrect (I) solutions per cluster. The colored sphere (green: high; orange: medium; red: acceptable; black: incorrect) in each boxplot indicates the CA-LRMSD of the cluster centroid.

**Figure S5:** T32; CA-LRMSD distribution of all clusters used for testing and training. Labels on the y-axis indicate the number of high (H), medium (M), acceptable (A) and incorrect (I) solutions per cluster. The colored sphere (green: high; orange: medium; red: acceptable; black: incorrect) in each boxplot indicates the CA-LRMSD of the cluster centroid.

**Figure S6:** T35; CA-LRMSD distribution of all clusters used for testing and training. Labels on the y-axis indicate the number of high (H), medium (M), acceptable (A) and incorrect (I) solutions per cluster. The colored sphere (green: high; orange: medium; red: acceptable; black: incorrect) in each boxplot indicates the CA-LRMSD of the cluster centroid.

**Figure S7:** T37; CA-LRMSD distribution of all clusters used for testing and training. Labels on the y-axis indicate the number of high (H), medium (M), acceptable (A) and incorrect (I) solutions per cluster. The colored sphere (green: high; orange: medium; red: acceptable; black: incorrect) in each boxplot indicates the CA-LRMSD of the cluster centroid.

**Figure S8:** T39; CA-LRMSD distribution of all clusters used for testing and training. Labels on the y-axis indicate the number of high (H), medium (M), acceptable (A) and incorrect (I) solutions per cluster. The colored sphere (green: high; orange: medium; red: acceptable; black: incorrect) in each boxplot indicates the CA-LRMSD of the cluster centroid.

**Figure S9:** T40; CA-LRMSD distribution of all clusters used for testing and training. Labels on the y-axis indicate the number of high (H), medium (M), acceptable (A) and incorrect (I) solutions per cluster. The colored sphere (green: high; orange: medium; red: acceptable; black: incorrect) in each boxplot indicates the CA-LRMSD of the cluster centroid.

**Figure S10:** T41; CA-LRMSD distribution of all clusters used for testing and training. Labels on the y-axis indicate the number of high (H), medium (M), acceptable (A) and incorrect (I) solutions per cluster. The colored sphere (green: high; orange: medium; red: acceptable; black: incorrect) in each boxplot indicates the CA-LRMSD of the cluster centroid.

**Figure S11:** T46; CA-LRMSD distribution of all clusters used for testing and training. Labels on the y-axis indicate the number of high (H), medium (M), acceptable (A) and incorrect (I) solutions per cluster. The colored sphere (green: high; orange: medium; red: acceptable; black: incorrect) in each boxplot indicates the CA-LRMSD of the cluster centroid.

**Figure S12:** T47; CA-LRMSD distribution of all clusters used for testing and training. Labels on the y-axis indicate the number of high (H), medium (M), acceptable (A) and incorrect (I) solutions per cluster. The colored sphere (green: high; orange: medium; red: acceptable; black: incorrect) in each boxplot indicates the CA-LRMSD of the cluster centroid.

**Figure S13:** T41; CA-LRMSD distribution of all clusters used for testing and training. Labels on the y-axis indicate the number of high (H), medium (M), acceptable (A) and incorrect (I) solutions per cluster. The colored sphere (green: high; orange: medium; red: acceptable; black: incorrect) in each boxplot indicates the CA-LRMSD of the cluster centroid.

 **Figure S14:** T53; CA-LRMSD distribution of all clusters used for testing and training. Labels on the y-axis indicate the number of high (H), medium (M), acceptable (A) and incorrect (I) solutions per cluster. The colored sphere (green: high; orange: medium; red: acceptable; black: incorrect) in each boxplot indicates the CA-LRMSD of the cluster centroid.

**Figure S15:** T54; CA-LRMSD distribution of all clusters used for testing and training. Labels on the y-axis indicate the number of high (H), medium (M), acceptable (A) and incorrect (I) solutions per cluster. The colored sphere (green: high; orange: medium; red: acceptable; black: incorrect) in each boxplot indicates the CA-LRMSD of the cluster centroid.

**Figure S16:** The predicted number of times a cluster is better vs. all other clusters (black cross) compared to the actual values (gray dots). The CA-LRMSD values on the x-axis are based on the cluster member with the lowest CA-LRMSD.
